# Surgical mechanical properties of perfused decellularized massive bone allografts: An comparative *in vivo test*

**DOI:** 10.1371/journal.pone.0322901

**Published:** 2025-06-02

**Authors:** Alicia Nuffer, Robin Evrard, Thomas Schubert, Benoit Lengelé, Alexis Veyssiere, Natacha Kadlub, Jean Boisson

**Affiliations:** 1 Service de chirurgie maxillo-faciale, chirurgie orale et reconstructrice, CHU de Caen, Caen, France; 2 Faculté de médecine, Université de Caen, Caen, France; 3 Laboratoire de Mécanique et de ses Interfaces, ENSTA, Palaiseau, France; 4 Secteur des Sciences de la Santé, Institut de Recherche Expérimentale et Clinique, Neuro Musculo Skeletal Lab (NMSK), Université catholique de Louvain, Bruxelles, Belgique; 5 Service d’orthopédie et de Traumatologie de l’appareil locomoteur, Cliniques Universitaires Saint-Luc, Bruxelles, Belgique; 6 Secteur des Sciences de la Santé, Institut de Recherche Expérimentale et Clinique, Pole de Chirurgie Expérimentale et Transplantation (CHEX), Université catholique de Louvain, Bruxelles, Belgique; 7 Secteur des Sciences de la Santé, Institut de Recherche Expérimentale et Clinique, Pole de morphologie (MORF), Université catholique de Louvain, Brussels, Belgium; 8 Service de chirurgie plastique, Reconstructrice et esthétique, Cliniques Universitaires Saint-Luc, Brussels, Belgium; 9 Université de Paris-Cité, UFR de médecine, Paris, France; 10 Service de chirurgie maxillo-faciale et Chirurgie plastique, APHP, Necker Enfant Malades, Paris, France; Semnan University, ISLAMIC REPUBLIC OF IRAN

## Abstract

Massive bone allograft decellularized by perfusion is a solution for large bone defect reconstructions. Perfusion-based decellularization offers a solution by removing cellular components while preserving the non-immunogenic matrix. This study evaluates the *in vivo* integration and mechanical properties of perfusion-decellularized bone grafts compared to “fresh-frozen” grafts, both before implantation and after explantation. Standardized porcine femoral grafts were categorized into non-irradiated, irradiated, and explanted groups, with half undergoing perfusion decellularization. Biomechanical tests, including screw pull-out test, compression, and 3-point bending test, were performed. Results indicate that while decellularization increases graft brittleness, Vickers indentation and compression tests showed no significant differences between groups. In our study, decellularization reduced the mechanical strength of allografts both before and after implantation. However, since the risk of rupture occurred only under mechanical loads exceeding the physiological range, perfusion-decellularized bone grafts remain a valid strategy for bone repair.

## Introduction

Reconstruction of large bone defects is a significant surgical challenge. Various techniques have been described in the literature depending on the patient’s condition and the underlying cause of bone loss. Autologous bone grafts and bone allografts, used alone or in combination with a flap [1–6], are not suitable for large defects, and tissue harvesting can result in disabling sequelae for the patient. These techniques are also associated with complications such as infection, implant failure, fracture, nonunion, necrosis, and poor functional outcomes [1,4–8].

To reduce these complications, vascularized allografts are a promising alternative. However, the face is one of the most immunogenic regions of the human body, requiring lifelong immunosuppressive therapy to prevent early or late allotransplant rejection [7–9]. This requirement imposes significant morbidity on patients, including serious infections, early-onset osteoporosis, organ failure, and immunosuppression-induced cancers [8].

Unlike composite allografts, bone allografts do not require immunosuppressive treatment due to the low cellularity of bone tissue. However, complications still occur, probably because bone allografts contain residual allogeneic biological tissue at the time of implantation. Tissue engineering offers a solution by providing three essential factors for bone integration: an osteoconductive scaffold, growth factors for osteoinduction, and cells with osteogenic potential. The scaffold material must meet certain criteria, including biocompatibility (the ability to support cellular activity without host toxicity), porosity (to facilitate cell ingrowth and nutrient diffusion), and sufficient mechanical strength for load transfer [10]. Biological scaffolds composed of extracellular matrix (ECM) can be derived from decellularized tissues or organs [11]. Decellularization involves the removal of all cells and immunogenic components from the donor tissue, resulting in an extracellular matrix that can be used as an allograft with reduced immune response. Consequently, a decellularized allograft could be recolonized by the recipient’s cells through the process of gradual substitution, thereby avoiding typical immunogenic complications while retaining the benefits of an autograft [12–14].

Decellularization techniques for small organs such as dermis and valves are well established, typically using immersion methods [15]. The current process of decellularization of bone fragments is performed by immersion [16].

Recently, new protocols involving perfusion decellularization, in which graft vessels are used to infuse decellularizing detergents, have allowed the production of larger and more complex scaffolds, such as bone, while preserving the architecture and vascular tree [17–20]. Evrard et al. demonstrated the efficacy of perfused-decellularized bone allografts in terms of absence of immunogenic materials and osteointegration [19,20].

For the reconstruction of critical-size bone defects, perfused decellularized bone could potentially overcome the limitations of bone repair. Bone repair strategies need to consider the mechanical properties of scaffolds. Unfortunately, the literature on the mechanical properties of decellularized bone is sparse [16,21–24], so it is unclear whether or not perfusion decellularization affects the mechanical strength of bone. For example, Anisimova et al [16] found that in dogs, decellularization decreased the mechanical properties of radius bones, while ulna in compression and humerus in flexion remained unchanged.

However, these studies used different decellularization processes based on immersion [16,21–24],

Our team conducted the first study comparing specific mechanical properties of decellularized vascularized bone grafts with native bone, which was published in a previous article [25]. Through various tests, it was found that decellularization with SDS (sodium dodecyl sulfate) did not adversely affect the mechanical properties of bone grafts, whereas decellularization with NaOH (sodium hydroxide) and H2O2 (hydrogen peroxide) appeared to weaken the mechanical integrity of the grafts under certain mechanical stresses. Conversely, the NaOH protocol resulted in better decellularization quality compared to the SDS protocol. A subsequent study by our team, focusing on the mechanical properties of NaOH perfusion-decellularized porcine femurs, confirmed these findings with an important addition: as long as the bone is subjected to physiological loading (within the range of its elastic phase), its mechanical properties are comparable to those of native bone [20].

The aim of the present study was to evaluate the mechanical properties of massive NaOH-perfused decellularized bone after irradiation and implantation in a porcine model for the repair of critical size bone defects, both before implantation and after explantation. In this study, as in [20], we focused on mechanical properties related to the surgical procedure and the postoperative period, such as screw pull-out strength and compressive strength. Meanwhile, we aimed to define a reproducible protocol to assess the mechanical properties of bone in a surgical context.

To our knowledge, the mechanical properties of ma ssive NA-OH perfused decellularized bone allograft, before and after implantation in a critically sized defect, have never been evaluated.

## Materials and methods

This study was conducted at the “Laboratoire de chirurgie expérimentale et transplantation (CHEX)”, part of the Institut de Recherche Expérimentale et Clinique (IREC) of the Université Catholique de Louvain, Brussels, Belgium, and at the Laboratoire de Mécanique et ses Interfaces in Palaiseau, France.

### Samples

#### Ethic rules.

All experiments were approved by the local ethics committee of UCLouvain (Brussels, Belgium) and were performed in accordance with the Belgian (Royal Decree, September 2004) and European Legislation (Directive-2010-63 UE), regarding animals used in experiments. Bones were obtained from pigs under the ethical committee number: 200/UCL/MD/027. All our samples were taken from Belgian Landrace pigs and samples were collected in the laboratory of the Institut de Recherche Expérimentale et Clinique de Louvain, Belgium.

#### Humane endpoints.

The humane endpoint was predefined in the study protocol to define the endpoint at which animals would be euthanized to reduce animal suffering. The humane criteria for euthanasia were: weight loss >20% of initial weight, food consumption <50%, occurrence of complications that could increase the animal’s discomfort (local or systemic infection, graft or plate fracture), pain not relieved by the analgesic regimen resulting in the animal’s lack of mobility, a marked reduction in food intake, or the occurrence of vocalizations.

#### The animal’s discomfort is assessed daily.

Food intake, wound progress, and behavior are recorded daily. Refinement is optimized primarily in terms of animal comfort: gels on pressure points during surgery, rubber mattresses, larger cages, games, contact with other animals, and a strict pre-established analgesic regimen. Analgesic protocol: - After premedication and induction of anesthesia for surgery, a continuous infusion of Temgesic (0.3mg/vial) was started immediately postoperatively via an atrial catheter. Two vials are injected into a 1L bag of physiologic fluid. The infusion will continue for 24–48 hours, depending on when the animal wakes up and its clinical progress. The continuous infusion will also help maintain the animal’s hydration status in the immediate post-operative period. Continuous infusion of Temgesic is continued for a minimum of 2 weeks at a dose of 2 vials/48h, then 1 vial/48h from the second week onwards. Each infusion may be supplemented with perfusalgan if necessary. The use of fentanyl is a last resort, but may be considered if the animal’s discomfort persists despite our basic regimen. NSAIDs (ketoprofen) are also administered at the following dosages 3mg/kg/day for 3 days. These treatments may be repeated with a break of at least the duration of the treatment.

All research personnel in contact with the pigs are certified with FELASA degree, special training in animal care or handling.

#### Harvesting grafts.

The experiments were performed with Aachen minipigs, from CER group (Rue du Point du jour, 8-6900 Marloie, Belgium; agreement number: LA2800428).

Bone grafts were harvested from femurs of juvenile female landrace pigs weighing approximately 40 kg (Ethical Agreements: 2020/UCL/MD/027 for harvested bones and 2021/UCL/MD/062 for surgical experiments). Female pigs were chosen, to facilitate the experimental model, as they are less aggressive, cleaner, and lighter to handle. Half of the femurs were decellularized by perfusion according to our previously described protocol [20].

#### Decellularization protocol.

The decellularization protocol used in this study was developed by Dr. Evrard’s team at the Institut de Recherche Expérimentale et Clinique (IREC) [19]. omplete decellularization of several femurs was achieved by perfusing porcine long bones with different solvents.

The protocol starts with dissection and cannulation of the nutrient artery of the bone. The bones are perfused with PBS (phosphate buffered saline) and heparin for 24 hours. The specimens are then immersed and perfused sequentially with acetone, NaOH, and H2O2 for a total of one week. A 24-hour wash with PBS is required between each step. Perfusion is performed with a peristaltic pump at an average flow rate of 6 mL/min.

**Fig 1:**
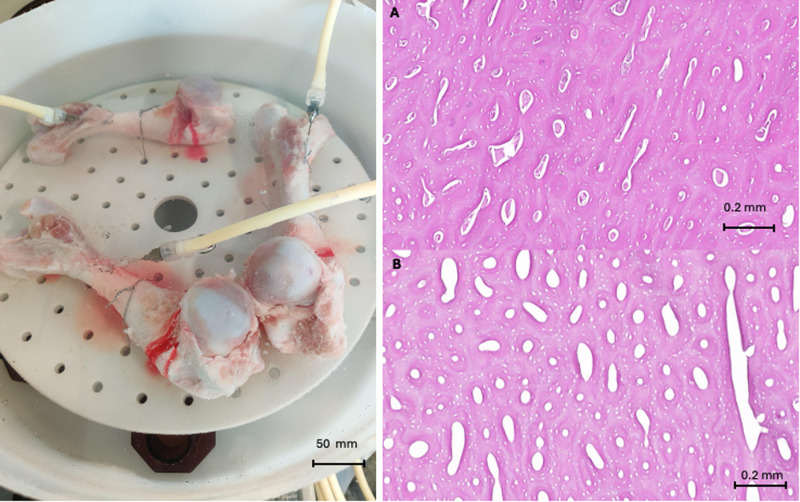
Decellularization by perfusion of pig femurs. Left: Initiation of the process with blood rinsing by heparinized PBS perfusion. Right: Histological control of decellularization by H/E staining. A: native bone filled with cellular components; B: decellularized bone freed from all cellular and nucleic components.

For rapid assessment of decellularization, diaphyseal and metaphyseal bone samples were harvested with an oscillating saw blade after removal of the diaphyseal allograft. These samples were then fixed in formaldehyde, decalcified, embedded in paraffin, and processed for conventional hematoxylin and eosin (H&E) histologic staining. Fig 1 shows the perfusion decellularization process and histologic controls.

#### Allografts preparation.

All allografts used in this study were obtained from porcine femurs. The samples were divided into two groups: one group underwent decellularization and the other remained untreated. The decellularized allografts were processed according to the described protocol and then stored at -80°C until transplantation. The size of the allografts was determined by calculating a bone defect size that would not heal spontaneously, defined as 1.5 times the diameter of the femoral diaphysis. To ensure consistency, 3D printed cutting guides were used to obtain the most reproducible specimens.

The allografts were further divided into three groups: two groups consisted of sixteen samples each (eight native and eight decellularized grafts, paired from the same femur) reserved for mechanical evaluation prior to transplantation, with one group undergoing irradiation treatment. The third group consisted of ten samples (five native and five decellularized grafts, paired from the same femur) intended for transplantation. These 10 specimens were sterilized by irradiation (30,000 Gy) prior to surgery.

#### Transplantation.

Five female mini-pigs, each at least two and a half years old and weighing approximately 50 kg, were selected to receive the bone grafts.

Female pigs were chosen to facilitate the experimental model as they are less aggressive, cleaner, easier to handle, and to have a homogeneous group of animals.

Treatment conditions for the pigs followed the guidelines of the Belgian Ministry of Agriculture and Animal Care. All procedures were approved by the local ethics committee for animal care at the Université catholique de Louvain (reference: 2021/UCL/MD/062).

**Fig 2:**
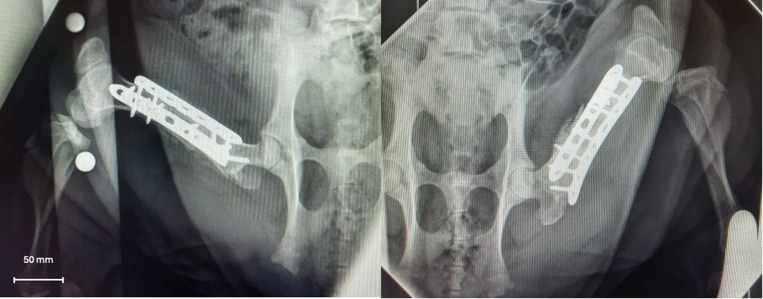
Postoperative radiographic controls: control of the correct position of the graft on each side (day 15).

The surgery, performed under general anesthesia, consisted of the creation of a critically sized bone defect followed by transplantation of the allografts. General anesthesia was induced by intramuscular injection of 6 ml/kg Zoletil 100® (Virbac, Carros, France) and 2 mg/kg Rompun® (Bayer, Leverkusen, Germany). Intravenous access was established through an ear vein. After tracheal intubation, anesthesia was maintained with isoflurane (Forene®, AbbVie, Wavre, Belgium) (0–1.5%) and oxygen. The critical bone defect size was created according to the animal model developed by Professor Schubert [26], defined as a defect size beyond which the porcine femur cannot spontaneously consolidate (critical defect = 1.5 times the diameter of the femoral shaft in the mid-diaphysis of one femur). As previously described, this size corresponds to 1.5 times the total diaphyseal diameter of the bone [27]. The bone was cut using an oscillating saw guided by corresponding 3D-printed cutting guides to ensure precise matching to the size of the bone grafts. Each pig received both types of allografts (decellularized and untreated), randomly assigned to either side, but always matched to the original donor. This approach allowed each pig to serve as both a control and a test, providing a comparative evaluation while reducing the number of pigs required. To ensure the stability of the construct, double conventional orthopedic plating was performed, allowing the pigs to walk immediately after surgery. Fig 2 shows the final image of the osteosynthesis.

#### Follow-up and explantation.

Clinical examination was performed daily with radiographs and CT scans every 2 weeks to assess bone consolidation (Fig 2). The outcome was poor in two pigs. The allografts had to be explanted after a few weeks. The allografts were infected on both sides and unusable for the study.

**Fig 3:**
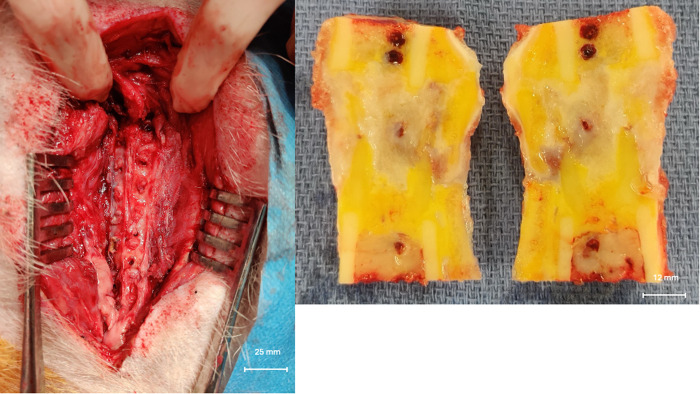
Allograft during explantation surgery. On the left, we can see the removal of the plate and screws just prior to allograft explantation. On the right, the allograft is explanted, cut in half, and stored at -80°C. The holes in the bone were made by the screws. This image shows an explanted decellularized allograft; macroscopically, a large area of bone remodeling (yellow) can be seen within the graft.

After three months, the three pigs were euthanized by lethal intravenous injection of T61® (Intervet, Belgium) under the same general anesthesia. The allografts were explanted and stored at -80°C (Fig 3). The allografts were cut into half cylinders 2.5 cm long and 2 cm in diameter. One half was used for histology/IHC and the other half for mechanical testing. Histology/IHC testing was the main outcome for a previous article [19]. Samples were kept frozen at -80°C until the mechanical testing was performed. Micro-CT scans were performed on these samples.

In the end, there were 38 samples (Table 1): 32 for preoperative samples (16 irradiated, 16 nonirradiated) and 6 for postoperative samples.

**Table 1:** Samples repartition.

	Group 1: Irradiated				Group 2: Non irradiated			
	Right femur		Left femur		Right femur		Left femur
Samples before transplantation	F1	Native		Decellularized	F1	Native		Decellularized
	F2	Native		Decellularized	F2	Native		Decellularized
	F3	Native		Decellularized	F3	Native		Decellularized
	F4	Native		Decellularized	F4	Native		Decellularized
	F5	Decellularized		Native	F5	Decellularized		Native
	F6	Native		Decellularized	F6	Native		Decellularized
	F7	Decellularized		Native	F7	Decellularized		Native
	F8	Decellularized		Native	F8	Decellularized		Native
	Group 3: Explanted (Irradiated before transplantation)							
Samples after transplantation			Right femur				Left femur	
	331		Native				Decellularized	
	336		Decellularized				Native	
	523		Decellularized				Native	

The letter F corresponds to femurs 1 through 8 of the four pigs. Groups 1 and 2 were not implanted, while group 3 was implanted in pigs and then explanted. 331, 336, and 523 are the pig identification numbers.

### Mechanical tests

Three mechanical tests were performed: screws extraction, compression and three-point bending. All tests were conducted at the LMI. We used a uniaxial tensile machine (34SC-5, Instron Corp., Illinois Tool Works Inc., Glenview, IL, USA) with a 5kN load cell (2519 series, Instron Corp., Illinois Tool Works Inc.).

**Fig 4:**
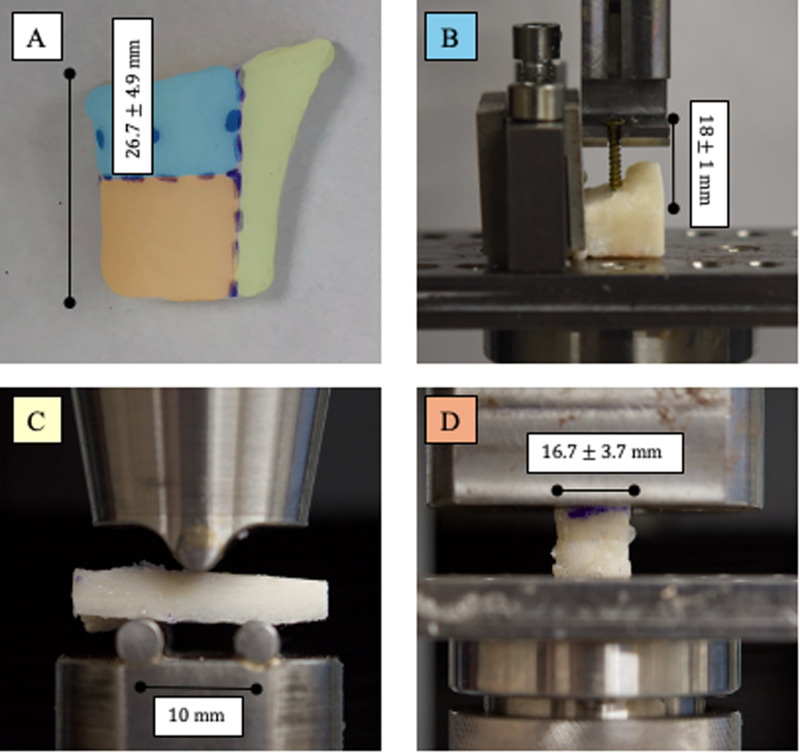
Mechanical tests. A: Locations of the various tests. Blue (B): three screw pull-out test, screw locations are represented by blue dots. Yellow (C): three-point bending test. Orange (D): Compression test.

For each specimen, we performed three pull-out tests on the proximal portion of the specimen, one compression test on the distal portion, and one three-point flexion test (Fig 4). To simplify the sample preparation, the tests were performed in this order.

The mechanical tests performed on the specimens aimed to evaluate their behavior in different surgical clinically relevant situations:

- Screw Pull-out Test: assesses screw retention during osteosynthesis
- Compression Test: Simulates bone load-bearing capacity during postoperative walking
- Three-Point Bending Test: Measures the bone’s flexural strength to postoperative trauma, such as falls or accidental impacts.

Due to anatomical constraints, non-standard specimens were used to optimize the number of tests performed while minimizing material loss. Also, the goal was to minimize specimen alteration by avoiding arbitrary cuts to achieve a perfectly rectangular shape. The dimensions of each specimen are provided in Supplementary Materials SD1 in S1 File. The tolerance for dimensional variation within a specimen was approximately ±1mm. We used 3D-printed cutting guides to ensure consistency between paired samples. For the compression test, special care was taken to ensure parallelism of the faces. In the three-point bending test, to ensure that the section within the loading span remained as constant as possible within the defined tolerance, the specimen was repositioned on the two supports. This adjustment helped minimize variations in cross-section in the critical bending region. Since, according to classical beam bending theory [28], the portions of the specimen outside the supports do not contribute to the bending behavior, any dimensional irregularities in those areas were considered negligible. Stability of each specimen on the two fixtures was ensured and the reported cross-sectional area is that of the critical bending region. Finally, to ensure consistency and comparability between native and decellularized specimens, each was shaped identically for a given test. It should be noted that every test was destructive.

#### Screw pull-out test.

The screw pull-out test, in which uniaxial traction is applied to an osteosynthesis screw inserted into bone, is commonly used in biomechanics to evaluate the ability of the bone to support osteosynthesis materials. This method has been used by orthopedic and dental teams to compare different types of screws as well as different bone anchorage locations [29–33]. To this study, a custom system was designed to generate axial tensile force only. Three tests were performed on each specimen according to the screw positions: central, lateral, and medial. The screws used were 18 mm, 2.3 mm diameter Stryker® screws (#50-23418). Holes were drilled into the bone with a 1.6 mm drill bit at 3000 rpm, penetrating 8 mm into the bone to traverse the entire cortical layer. The screw was then inserted to a depth of 6 mm, leaving 12 mm of the screw exposed (Fig 4B). This length of penetration allows the screw to have a good grip on the cortical bone. Tensile test was performed at a constant rate of 1 mm/min until the measured load dropped by 40%, which corresponds to the bone fracture around the screw thread. Force (N) and displacement (m) data were recorded, and the work required to extract the screw, expressed in Joules (J), was evaluated as the area under the force-displacement curve from the origin to the maximum force.

#### Compression test.

The last section of the bone specimen was used for compression testing. Compression testing is a standard method for mechanical characterization of materials and is preferred over tensile testing for small specimens [34–36].

For compression testing, it is essential that the specimens have two parallel and smooth surfaces for accurate results [36]. To achieve this, a custom 3D-printed cutting guide was designed. The specimen was secured in the cutting guide, and a precision cutting machine (Buehler Isomet 1000) equipped with a diamond disk (152 mm diameter, 0.5 mm thickness) was used to make the first cut. The entire system was then inverted to make a second cut parallel to the first (152 mm diameter, 0.5 mm thickness) was used to make the first cut (SD1 and SD2 in S1 File). This method was chosen over polishing techniques to minimize excessive bone loss. Parallelism was verified using a caliper to ensure minimal deviation between the opposing faces.

The compression test was started with 10 compression cycles at 0.3% deformation at a rate of 0.01 mm.$s^{-1}$. These parameters were set according to recommendations found in the literature [37]. A compression test was then performed at the same rate. The test was stopped when the local maximum was reached, followed by a decrease in force of more than 20% (Fig 4D).

The force (F) and displacement (l) data were extracted. The force data were then converted to nominal stress (σ), which is defined as the force normalized to the initial sample section $S_{0}$ [25],

$\sigma =\frac{F}{S_{0}}.$

Here, the nominal stress is in Pascals (Pa), the force is in Newtons (N) and $S_{0}=w_{0}\times t_{0}$ is the approximated initial surface area in square meters (m²), where $w_{0}$ and $t_{0}$ are respectively the initial width and thickness. The paired specimens, which reasonably have the same dimensions (see Table 2), made the comparison between native and decellularized specimens relevant. The strain is defined as in [25],

**Table 2:** Sample dimension for compression and three-points bending tests.

	Compression			Three-points bending test		
	Length (<<Eqn2>>	Width (<<Eqn3>>	Thickness (<<Eqn4>>)	Length(<<Eqn5>>)	Width (<<Eqn6>>)	Thickness (<<Eqn7>>
Irradiated	14.4 ±2.4	14.9 ±4.5	6.4 ±2.4	26.7 ±4.5	11.2 ±2.2	5.3 ±1.2
Non-irradiated	13.6 ±2.3	16.7 ±3.7	6.8±1.2	26.7 ±4.9	11.2 ±1.7	5.3 ±1.5
Explanted	14.5 ±1.9	20.7 ±4.9	11 ±2.8	21.6 ±5.3	11.4 ±2.7	7.4 ±2.4

The averaged length, width and thickness are reported in mm with their corresponding standard deviations.

$\varepsilon =\frac{\Delta l}{l_{0}},$

where $\Delta l=|l-l_{0}|$ the variation of the distance between the two plates measured during the compression test and $l_{0}$ is the initial distance between the two plates, both in meters (m).

The tangent Young’s modulus E (MPa) is calculated from the slope of the linear part - i.e., approximatively in the strain range of 1.5–2.5% for the irradiated and non-irradiated groups, and in the range of 2–4% in the explanted group - of the compression curve (σ,ε) for the native and decellularized samples across the three groups as in [16]:

σ=Eε.

#### Three-points bending test.

The area of the specimen damaged by the screw pull-out tests was removed and the remaining specimen was divided into two sections. One section was retained in its full length for three-point bending tests, while the other section was prepared for compression tests.

During the three-point bending tests (Fig 4C), the load was progressively increased until the bone fractured [35,38,39]. A custom jaw was designed with a distance of 10 mm between the two fixtures to ensure accurate testing of all specimens. Due to the small size of the specimens, the jaw was specifically designed to provide accurate and reproducible results (see Table 2). Tests were performed at a constant rate of 1 mm/min until the measured load dropped by 40%, corresponding to bone fracture.

From these tests, the modulus of rupture is calculated to evaluate the bone’s flexural strength. Approximating the bone cross section as rectangular, we defined stress $\sigma_{B}$ as follows [40]:

$\sigma_{B}=\frac{3F_{B}H}{2W_{0}T_{0}^{2}},$

where $F_{B}$ is the maximum measured force (in Newton), H is the distance between the two fixtures ($H=10^{-2}$ m), $W_{0}$ is the initial width and $T_{0}$ is the initial thickness of the specimen reported in Table 2. The bending strain $\varepsilon_{f}$ is also defined approximating a rectangular cross-section [40]:

$\varepsilon_{f}=\frac{6h}{L^{2}},$

Due to the lack of standardized specimens, we attempted to maintain consistent shapes within each experimental category to allow for meaningful comparisons between specimens. These results were compared with values reported in the literature.

### Statistical analysis

Statistical analyses were performed using Python 3.11.3 software. For each test, the decellularized specimens were compared to the corresponding native specimens. For each mechanical parameter, the ratio between native and decellularized results was calculated for each matched sample. The number of samples was too small to calculate a p-value, so the results are presented with a standard deviation value [41].

## Results

### Decellularization step

#### Samples.

Three animals were euthanized because they reached the end of the experiment. The entire animal study lasted 3 months. Two animals were euthanized because the human endpoint was reached: weight loss >20% and local infection of the surgical site. They were euthanized within 1 day by simultaneous intramuscular injection of Zoletil (6 mg/kg) and Rompun (2 mg/kg), placement of a peripheral atrial catheter for injection, and slow lethal intravenous injection of T61 (1 ml/5 kg).

Ten femurs treated with the cannulation-perfusion technique and the NaOH/H_2_O_2_ decellularization protocol were evaluated. Compared to native bone, no cells were detected by histology. The intramedullary spaces were completely empty and washed of all bone marrow and blood cells. This conventional histological evaluation confirmed that the perfusion decellularization protocol was performed correctly.

#### Macroscopic and micro-CT scan analyses.

**Fig 5:**
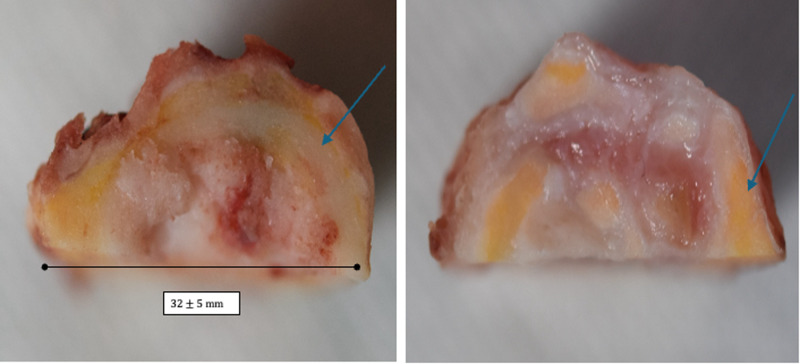
Matched allograft from the explanted group (Pig 336). On the left, the native explanted specimen and on the right, the decellularized explanted specimen. Blue arrows indicate what may be bone remodeling areas corresponding to osteoid matrix, which is present in greater amounts in the right image.

Macroscopic analyses are of particular interest for explanted specimens. Explanted allografts submitted for mechanical testing were whole allografts harvested during surgery. Removal of soft tissues was not possible because the allografts were composed of bone tissue and fibrous healing tissue. The proportion of mineral tissue appeared to be lower in decellularized allografts than in native allografts (Fig 5). The yellow areas shown in this figure may represent the volume of osteoid matrix that hasn’t yet mineralized.

Based on these macroscopic findings, micro-CT images were analyzed to determine the volume of mineralized bone in relation to the volume of non-mineralized tissues such as fibrosis, osteoid matrix, muscle, spinal cord, or periosteum. Results were presented in Table 3.

**Table 3:** Results of micro-CT-scan analyses for the explanted group.

Explanted samples		Percentage of bone
331	Right Native	41.6%
	Left Decellularized	16.3%
336	Right Decellularized	22.1%
	Left Native	34.9%
523	Right Decellularized	27.2%
	Left Native	39.0%

The table showed the percentage of mineralized bone for each sample.

### Mechanical tests

#### Screw pull-out test.

**Fig 6:**
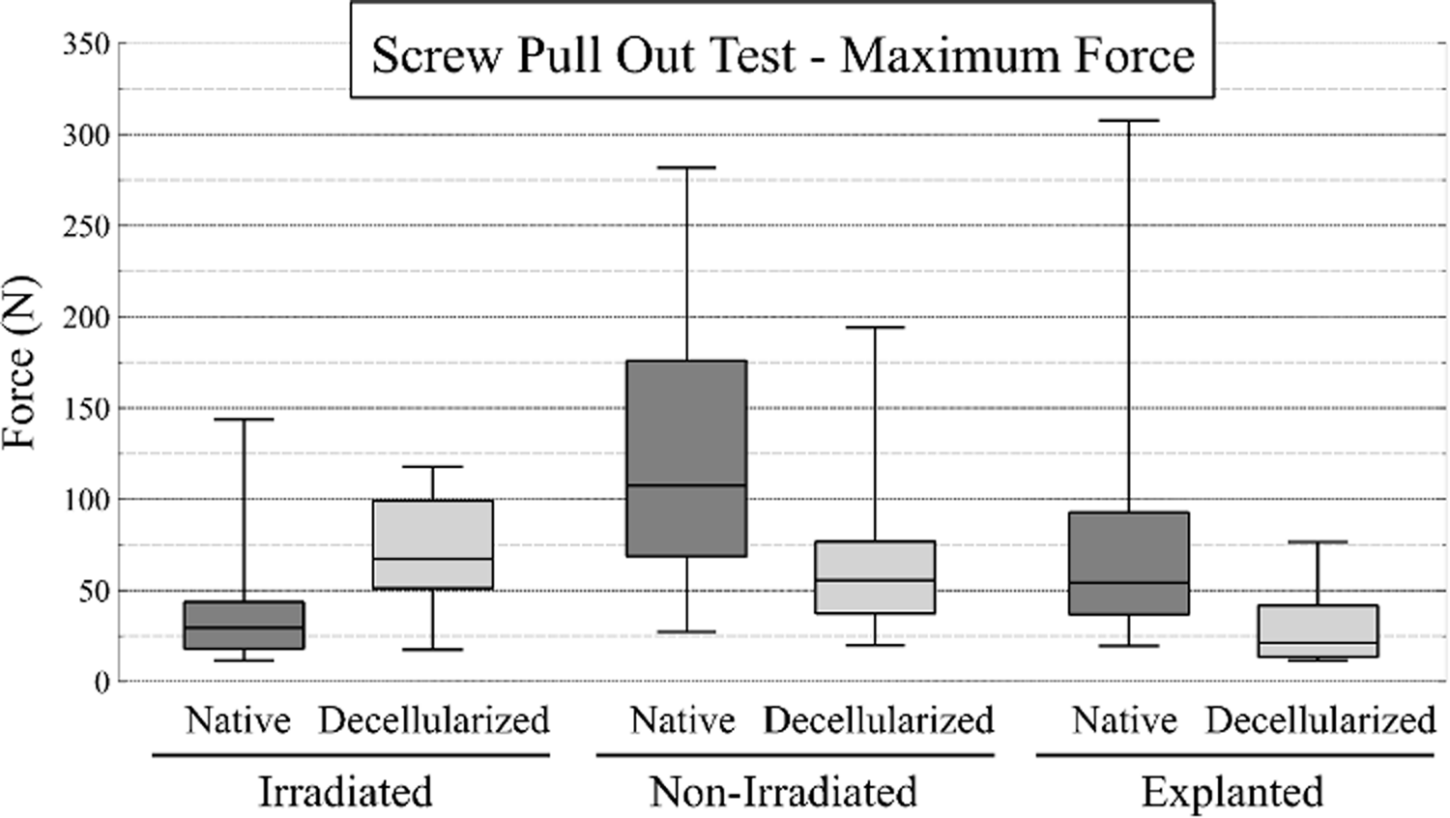
Results of the screw pull-out test for the extraction force. The box plots illustrate the maximum force (N) for the native and decellularized samples across the three experimental groups.

**Fig 7:**
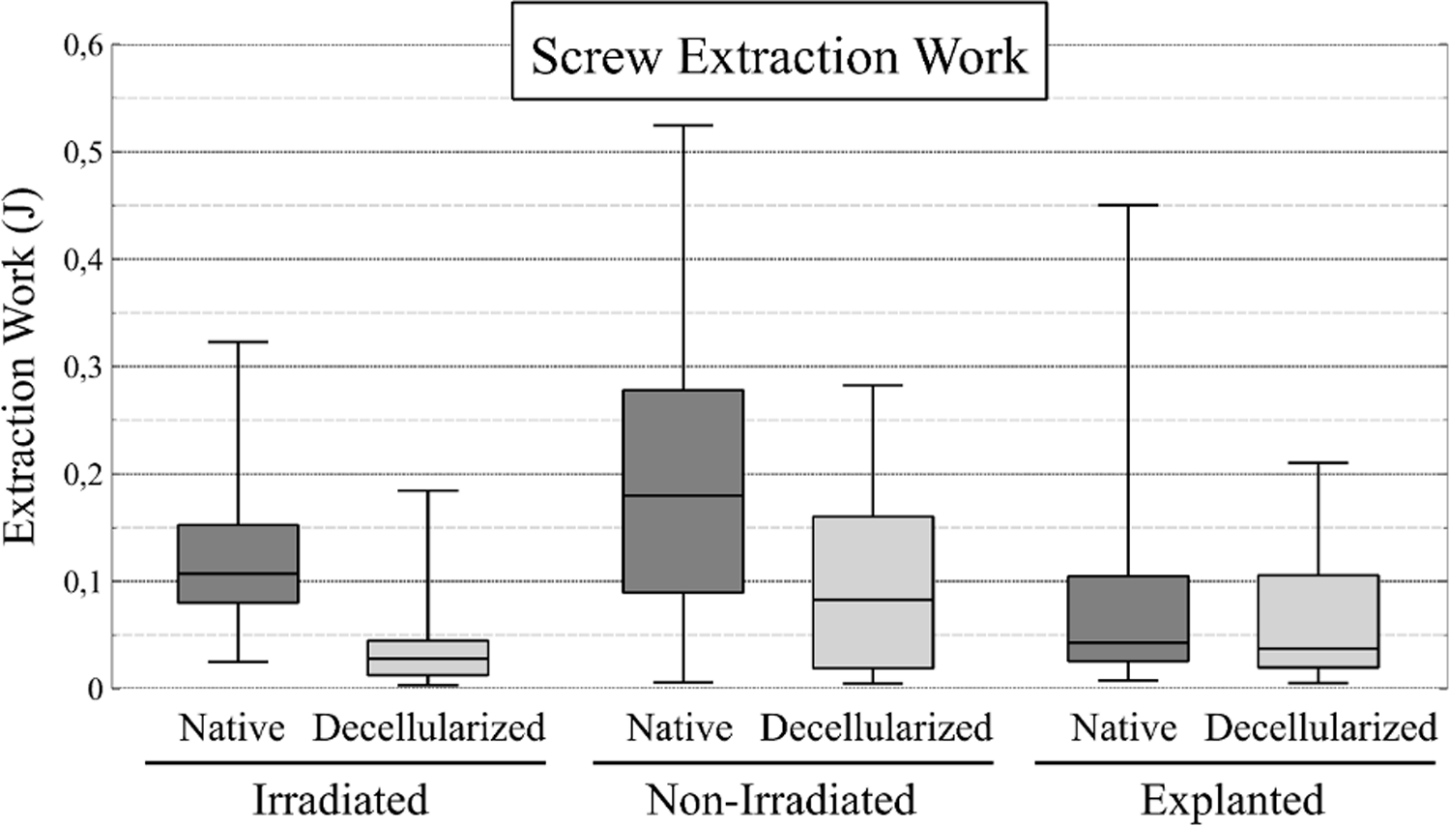
Results of the screw extraction work. The box plots illustrate the data pertaining to screw extraction (J) for the native and decellularized samples across the three groups. The work is calculated from the area under the force-displacement curve.

A total of 48 tests were performed for the irradiated group, 48 for the nonirradiated group, and 18 for the explanted group. Samples size are detailled in SD1 in S1 File. Some values were excluded from the analysis: one native and one decellularized specimen from the irradiated group were discarded due to manipulation errors during testing, and one native specimen from the explanted group was excluded due to damage sustained after the lateral and central screw extraction tests. The results are shown in Figs 6 and 7.

The mean maximum forces were 35 ± 28 N for native specimens and 76 ± 34 N for The mean maximum forces were 35 ± 28 N for native specimens and 76 ± 34 N for decellularized specimens in the irradiated group, and 130 ± 73 N for native specimens and 62.6 ± 35 N for decellularized specimens in the nonirradiated group. In the explanted group, the mean values were 87 ± 93 N for native specimens and 31 ± 23 N for decellularized specimens (the standard deviation of the first value tends to misinterpret statistical analysis). The coefficients were calculated for each matched sample and for each measured parameter (maximum force and extraction work) using the ratio of native to decellularized values. The mean and standard deviation (SD) of the ratios is presented in Table 4.

**Table 4:** Results of pull-out tests.

	Maximum Force ratio	Screw extraction work ratio
	Mean Native/Decellularized <<Eqn50>> SD	Mean Native/Decellularized <<Eqn51>> SD
Irradiated	3.2 ± 2.3	3.0 ± 2.5
Non-irradiated	2.1 ± 1.6	1.3 ± 1.1
Explanted	2.8 ± 2.3	1.2 ± 1.0

The table presents the means and standard deviations (SD) of the native/decellularized ratio for extraction force and screw extraction work.

#### Compression-test.

**Fig 8:**
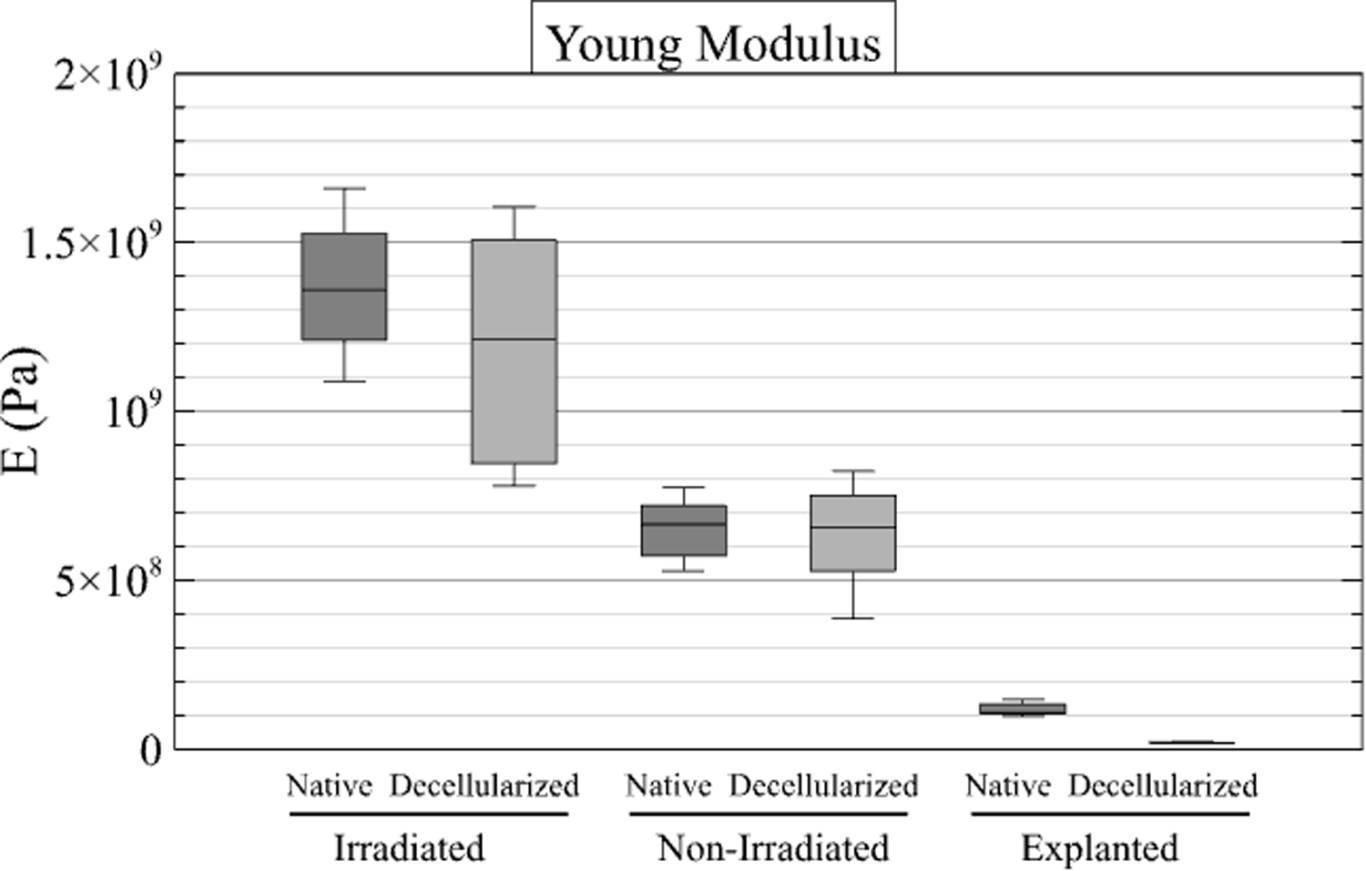
Results of compression tests. Box plots illustrate the tangent Young’s modulus (MPa) measured in the linear part of the curve for the native and decellularized samples across the three groups.

**Fig 9:**
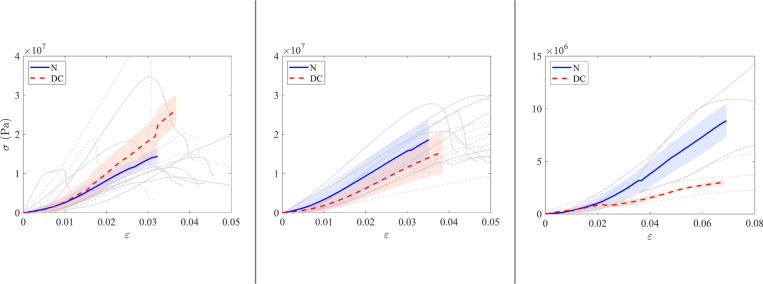
Compression test curves. The graph shows the stress-strain curves for the three groups: irradiated (left), non-irradiated (middle), explanted (right). Each curve represents a specimen from the explanted group. The solid blue curves represent native samples, while the dashed red curves represent decellularized samples. The thick curves correspond to the averaged curves. Tangent Young’s modulus is derived from the linear portion of the curve.

Compression tests were performed on all specimens except one native and one decellularized matched specimen in the irradiated group (F6) due to the presence of pre-existing fractures. Finally, 14 compression tests were performed in the irradiated cohort (7 native and 7 decellularized), 16 tests in the nonirradiated cohort (8 native and 8 decellularized), and 6 tests in the explanted cohort (3 native and 3 decellularized). The results are shown in Figs 8 and 9.

The mean values of tangent Young’s modulus were 1374 ± 305 MPa for the native specimens and 1192 ± 452 MPa for the decellularized specimens in the irradiated group, 651 ± 132 MPa for the native specimens and 6046 ± 233 MPa for the decellularized specimens in the irradiated group, and 122.6 ± 30.6 MPa for the native specimens and 30 ± 4 MPa for the decellularized specimens in the explanted group. The mean values of ultimate compressive strength were 16.3 ± 4.0 MPa for the native specimens and 19.0 ± 6.1 MPa for the decellularized specimens in the irradiated group, 23.9 ± 6.3 MPa for the native specimens and 19.0 ± 4.7 MPa for the decellularized specimens in the non-irradiated group, and 12.0 ± 4.1 MPa for the native specimens and 8.5 ± 9.2 MPa for the decellularized specimens in the explanted group. To facilitate a comparative analysis of native and decellularized allografts, the mean ratio of native to decellularized values was calculated for tangent Young’s modulus and compressive strength in the three experimental groups. The results are shown in Table 5.

**Table 5:** Results of compression tests.

	Tangent Young Modulus ratio	Max Compression Stress ratio
	Mean Native/Decellularized <<Eqn58>>SD	Mean Native/Decellularized <<Eqn59>>SD
Irradiated	0.94 ± 0.41	0.95 ±0.37
Non-irradiated	1.49 ±0.36	1.32 ±0.42
Explanted	6.25 ±2.28	2.23 ±1.83

The table displays the means and confidence intervals for the native/decellularized paired ratio of the tangent young modulus and of the maximum compression stress. SD = standard deviation.

#### Three-points bending test.

**Fig 10:**
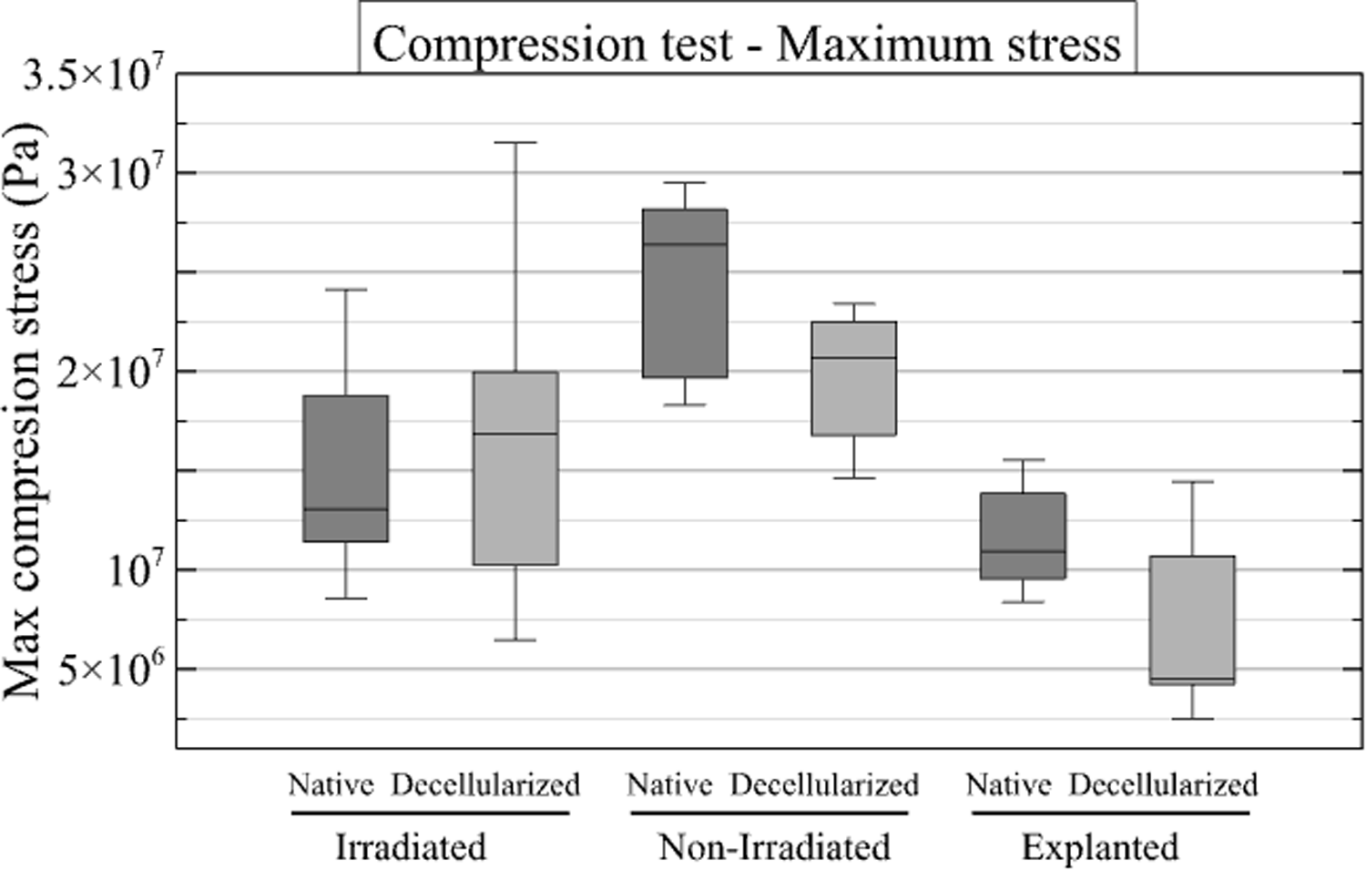
Results of compression tests. Box plots illustrate the maximum compression stress (MPa) for the native and decellularized samples across the three groups.

**Fig 11:**
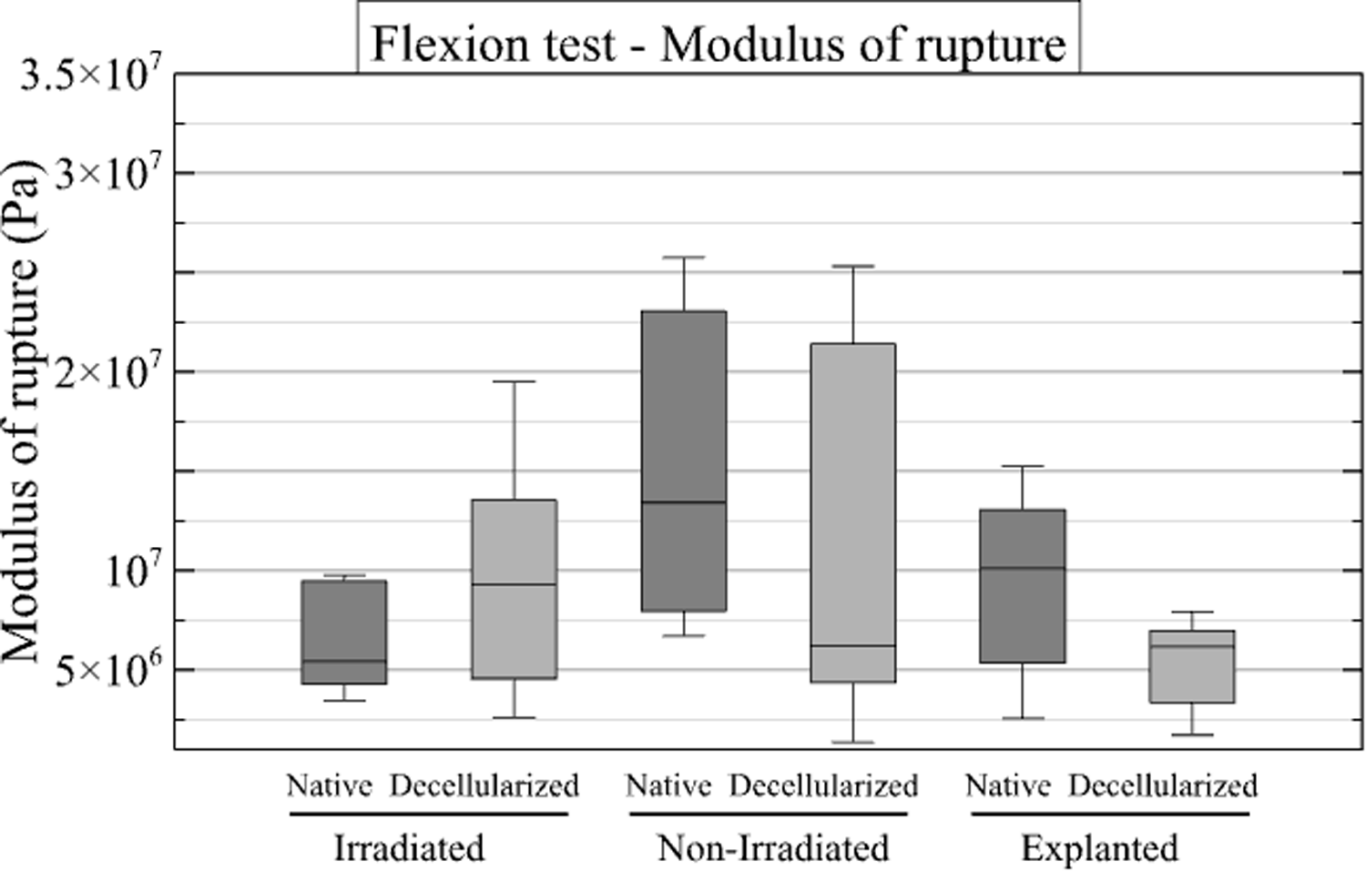
Results of the 3-points bending test for modulus of rupture. The box plots illustrate the modulus of rupture for the native and decellularized samples across the three experimental groups.

**Fig 12:**
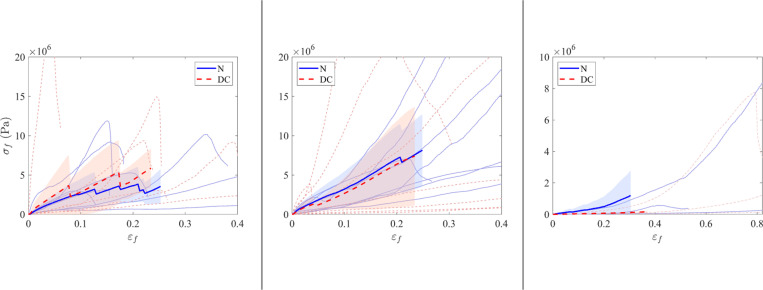
Curves of 3-point bending tests for the irradiated group (left), the non-irradiated group (center) and the explanted group (right). The bending stress along the bending strain $\varepsilon_{f}$ is plotted. Each curve represents one specimen. The solid blue curves represent native samples, while the dashed red curves represent decellularized samples. The thick curves correspond to the mean curves and the light areas are the standard deviation around the corresponding mean curve.

Subsequently, three-point bending tests were conducted on 38 samples, comprising 19 native and 19 decellularized samples. The results are presented in Figs 10–12, SD1 in S1 File.

The modulus of rupture was 6.6 ± 3.3 MPa for the native samples and 11.1 ± 3.4 MPa for the decellularized samples in the irradiated group. In the non-irradiated group, the modulus of rupture was 16.2 ± 10.1 MPa for the native specimens and 13.3 ± 13.6 MPa for the decellularized specimens. In the explanted group, the mean values were 8.9 ± 6.7 MPa for native specimens and 4.8 ± 1.1 MPa for decellularized specimens.

The coefficients from the ratio of native to decellularized samples were calculated for both the maximum force and the work required for bending. The mean values with their standard deviations (SD) are presented in Table 6.

**Table 6:** Results of 3-point bending tests.

	Modulus of rupture ratio
	Mean Native/Decellularized <<Eqn67>>SD
Irradiated	1.56 ±1.62
Non-irradiated	2.65 ±2.58
Explanted	10.6 ±16.9

The table displays the means and confidence intervals for the ratio of native to decellularized samples for both the maximum force and work for the three-point bending test. SD = standard deviation.

## Discussion

In this study the authors evaluate the mechanical properties of perfused-decellularized massive bone grafts compared to “fresh-frozen” grafts before implantation and after explantation.

The decellularization protocol used in this study was based on NaOH and H₂O₂, as previously described [27]. This protocol was previously used in a comparative study of two different decellularization methods [25]. The SDS-based protocol appeared to result in less significant decellularization than the NaOH and H₂O₂-based protocol. The results showed a reduction in DNA content, indicating less inflammatory and thrombogenic recellularization at the histological and immunostaining levels [42]. It is noteworthy that this protocol has been extensively studied *in vitro*, both from a biological [43] and mechanical [20] perspective. However, it has yet to be evaluated after an *in vivo* period.

The objective of this work was to mechanically simulate the solicitation of a bone after surgical implantation. Therefore, an extensive mechanical study was not performed. Also, since we wanted to compare native and decellularized bone, we focused on the performance ratios in the following. First, however, we can compare the raw results with the literature. The tangent Young’s modulus from the compression test is approximately 1 GPa for both irradiated and non-irradiated bones, while the ultimate compressive stress and modulus of rupture (around 10 MPa) are lower than the values reported in comparable literature - i.e., approximately 7 GPa for the tangent Young’s modulus in [16] and around 100 MPa for the modulus of rupture in [22,23]. This is probably due to the origin of the samples (juvenile female landrace pigs). However, focusing on the comparison of native and decellularized samples in the non-irradiated group, results were obtained that were consistent with those reported in the literature, in particular those of a previous study [25]. The results of the screw pull-out test and the compression test indicate that decellularized allografts have reduced mechanical strength compared to native specimens. The sodium hydroxide and hydrogen peroxide decellularization protocol used in this study appears to affect the mechanical properties of bone. A review of the literature reveals a lack of consensus regarding the results of soft tissue studies. In fact, Van Steenberghe and colleagues suggest that a NaOH-based process does not affect biomechanical properties [44]. In another study, the same authors demonstrated a significant mechanical difference in NaOH-decellularized tissue [45]. The mechanical properties of decellularized bone treated with NaOH and H₂O₂ were previously evaluated [25]. A series of tests were performed during the study, including screw pull-out, compression, Vickers indentation, and 3-point flexure. The results of the three-point flexure and pull-out tests indicated that the decellularized specimens exhibited increased susceptibility to fracture [25]. The results are comparable to those obtained in our previous study in terms of maximum force and work for the pull-out test and the 3-point bending test. In contrast, the Vickers indentation and compression tests showed no statistically significant differences between the experimental groups. This last statement confirms the results presented in a previous publication [20] that the perfusion-decellularization protocol will affect the biomechanical properties of the bone, but only when mechanical loads exceed the physiological stress range (as implemented in our tests).

The discrepancy between the native and decellularized samples for bone materials may be attributed to the use of NaOH, which has been observed to cause demineralization of the cortical bone by dissolving the bone mineral [46,47]. As shown by Su et al. in their article [46], the application of NaOH resulted in a significant reduction in the weight percentage of both mineral and organic material within the bone. *A Contrario,* H₂O₂ has minimal or no effect on bone composition. De Paula et al [48] demonstrated a demineralizing effect of H2O2, but this process is superficial and may not be a significant factor in explaining the loss of mechanical strength after decellularization.

The base treatments, NaOH and KOH, resulted in a reduction in the weight percentage of both mineral and organic material in bone [20]. Despite these adverse effects of NaOH, this method of perfused decellularization offers a better efficiency than H2O2 methods [19,20]. In addition, a reduction in mechanical stress at a non-physiological load was observed [25]. Finally, the perfused-decellularized graft is an osteoinductive scaffold whose future is to be recolonized and replaced by cells from the recipient, which will have the properties of native bone. In this context, temporary relative of weakness, could be accepted to avoid infection and rejection due to persistence of immune materials.

The irradiated group was exposed to 30 kGy of gamma radiation applied to all native and decellularized specimens. The decellularized specimens showed reduced screw pull-out strength in the screw pull-out test compared to the native specimens. However, the decellularized samples exhibited a higher tangent Young’s modulus in the compression test than the native samples. These different results can be attributed to the cumulative effects of irradiation and the decellularization protocol. As noted above, the NaOH-based protocol was associated with the formation of cracks on the bone surface [46]. In addition, radiation has been shown to have a negative effect on the mechanical properties of bone [49]. The combination of these two protocols may explain these results. In the three-point bending test, the irradiated bone samples showed less effect of decellularization, with the resulting data more similar to that of the native samples. A comparison of the irradiated and non-irradiated groups showed that the average ratio of native to decellularized samples was higher in the irradiated group, indicating a greater difference between the two groups. This discrepancy is attributed to the effects of irradiation. The detrimental effects of gamma irradiation on bone mechanical properties have been extensively documented in the scientific literature and the underlying mechanisms are well understood. In general, the elastic behavior of cortical bone tissue remains unaffected, while the plastic properties are significantly reduced. The degradation of the plastic phase results in a loss of bone tissue ductility, rendering gamma irradiated bone tissue brittle [50–52]. Gamma irradiation is a common method of sterilizing allografts prior to transplantation. A standard recommended dose of 25–35 kGy is required to inactivate the HIV virus in bone allografts [49]. However, the radiation dose required to achieve a sterility level of 10-⁶ has been reported to be as high as 89 kGy [53], which would result in significant damage to the mechanical properties of the bone. Accordingly, an irradiation dose of 30 kGy was chosen for this protocol in accordance with current recommendations. However, Currey et al [54] observed that even the standard dose of 25 kGy significantly reduces bone strength, with only minor changes in elastic modulus but significant reductions in plastic behavior between bones irradiated at 29.5 kGy and control samples. For example, the flexural strength of bones irradiated at the standard dose was approximately 20–30% lower than that of controls, and irradiation resulted in a reduction in work-to-fracture of more than 70% [54]. These results do not fully explain the observed increase in tangent Young’s modulus. However, it is reasonable to hypothesize that the combination of irradiation and the damage induced by the NaOH-based decellularization process may contribute to an increase in bone stiffness.

The focus of this study was on the screw pull-out test and the compression test, which allowed us to conclude that the native specimens had greater mechanical strength than the decellularized specimens. The mechanical tests performed on the explanted group yielded unexpected results. The tangent Young’s modulus of the explanted allografts was found to be four times higher in the native group than in the decellularized group. This discrepancy may be attributed to the composition of the allografts. Macroscopic analysis of the explanted allografts showed a reduced volume of mineralized bone in the decellularized samples compared to the native samples. The osteoid matrix that typically forms during the healing process is known to be less stiff than bone because it is not yet mineralized, resulting in a lower tangent Young’s modulus [55]. It is noteworthy that the three-point flexure test results show a greater flexural strength in the decellularized specimens compared to the native specimens. This discrepancy may also be explained by the distribution of bone remodeling areas, including osteoid matrix, which may not be present in the small samples used for this test. As a result, the discrepancies between the groups may not be as obvious as for other measurements.

Macroscopic and micro-CT scan analysis revealed incomplete ossification in both native and decellularized specimens, with this problem being more pronounced in the decellularized specimens. This lack of allograft consolidation could be attributed to the relatively short explantation time. Indeed, Aranguren et al. reported that bone consolidation after massive bone allograft transplantation requires at least six months [56]. This delay is confirmed by several studies in the literature [57,58]. In the present study, however, the allografts were explanted after only three months postoperatively. This shortened period did not allow sufficient time for adequate consolidation of both native and decellularized bone allografts. Furthermore, biological analysis of the explanted allografts revealed a significantly increased amount of bone remodeling in the decellularized allografts. This finding supports the hypothesis that decellularized allografts are more susceptible to bone remodeling and integration. As a result, explantation of the grafts only three months after surgery resulted in less solid implants due to the presence of a greater amount of non-mineralized osteoid matrix, as confirmed by micro-CT imaging. The native allografts, which have not undergone the same degree of integration, are dependent on the donor tissue, which may remain in the patient as dead bone. In contrast, decellularized allografts undergo active remodeling with the goal of replacing the donor bone extracellular matrix (ECM) with living autologous bone [57,58].

Three mechanical tests were performed to provide a more complete assessment of the bone properties. Of note, the screw pull-out test was critical in evaluating the mechanical strength of the allograft when exposed to osteosynthesis material, which is of paramount importance in allograft fixation. Accordingly, custom devices were designed to perform all three tests. However, the screw pull-out test required the entire specimen to be securely fixed to prevent movement during the test, which could have biased the results. It is possible that the jaws used in this process may have damaged the specimens, which could have affected their suitability for subsequent testing. For example, three specimens in the irradiated group, including two matched specimens, were unable to undergo the compression test or the medial screw pullout test because they developed fractures during the testing process. In addition, the need to excise specimens for the compression and three-point bending tests further reduced the available specimen size. Compression tests were performed on specimens with a minimum height of 10 mm. However, Athanasiou and colleagues have demonstrated the feasibility of performing compression tests on small specimens [35].

To avoid bias, custom cutting guides ensured that the samples used were of similar dimensions, but the sample size itself was relatively small (2.5 cm in length and 2 cm in diameter). To account for inter-individual variability, a paired analysis was performed in which each decellularized sample was paired with a native sample taken from the same bone. The goal of this approach was to minimize inter-sample variability. However, the limited number of samples prevented a complete analysis of the mechanical behavior. Nevertheless, all tests were performed according to standardized procedures, ensuring that each sample was subjected to the same systematic error and allowing comparisons to be made.

The number of samples in each group - irradiated, non-irradiated and explanted - limits the ability to make direct comparisons between groups. However, the process of matching the samples allows for a more direct comparison. The mechanical strength of the decellularized specimens showed a more pronounced change after surgery compared to the non-transplanted specimens. On the other hand, the 3-point bending test yielded different results, possibly due to the different proportions of bone in the allografts after transplantation.

This study, explore for the first time in literature the surgical mechanical properties of massive perfused-decellularized and sterilized bone graft, which is essential before human clinical use. The outline of this study should be exposed to improve a subsequent experiment before human use. A next protocol should evaluate the bone graft after 6 months implantations to access the bone integration, a larger specimen should be tested, and more pigs should be included. Further studies in animal models are needed to gain a deeper understanding of our findings, a more comprehensive and thorough investigation is imperative, with particular emphasis on the explanted cohort. This would require the inclusion of a larger sample size of allografts implanted and subsequently explanted. It is essential to maintain the method of matching native samples with decellularized samples as this provides a more reliable basis for comparison. In addition, it is imperative to extend the duration of allograft presence *in vivo* to allow sufficient time for bone consolidation. In addition, it may be beneficial to perform a comparative analysis of *in vivo* recellularization after decellularization using different protocols. For the future, it would be prudent to consider alternative sterilization methods other than irradiation to avoid any potential compromise of the mechanical integrity of the allografts prior to transplantation.

### Limitations

In this study, we identify some limitations that could affect the conclusion of this research.

- The most important limitation is the duration before explantation, which could have affected the biomechanical results. As shown in Fig 5 and Table 3, bone was not observed in the whole specimen, which could explain the variability of the results.
- The number of specimens may be insufficient from a statistical point of view.
- By design, the tests do not investigate the full range of mechanical properties, but only those that could affect the surgical process.

## Conclusions

Mechanical tests carried out on native and decellularized samples revealed significant differences depending on experimental conditions (irradiated, non-irradiated, explanted).

- In screw pull-out tests, native samples showed a higher extraction force than decellularized samples, with an average native/decellularized ratio of 3.2 ± 2.3 for the irradiated group, 2.1 ± 1.6 for the non-irradiated group, and 2.8 ± 2.3 for the explanted group.
- Compression tests revealed a reduction in the tangent Young’s modulus for decellularized specimens in the explanted group, with an average native/decellularized ratio of 6.25 ± 2.28, compared with 0.94 ± 0.41 for the irradiated group and 1.49 ± 0.36 for the non-irradiated group. For maximum compressive stress, the native/decellularized ratio was 0.95 ± 0.37 for the irradiated group, 1.32 ± 0.42 for the non-irradiated group and 2.23 ± 1.83 for the explanted group.
- Finally, three-point bending tests showed that the strength of decellularized samples was lower in the explanted group, with a native/decellularized ratio of 10.6 ± 16.9, whereas this ratio was 1.56 ± 1.62 for the irradiated group and 2.65 ± 2.58 for the non-irradiated group.

This weakness occurred over physiological constraint and should be limited in time (physiological bone remodeling). Mechanical strength is particularly affected when decellularization is performed with irradiation; these findings should lead us to consider a sterilization method other than irradiation.

NA-OH irradiated perfusion-decellularized massive bone is effective in avoiding immunogenic and infectious rejection, despite an acceptable and temporary reduction in bone mechanical strength compared to native bone. Decellularization affects some mechanical properties of bone. Since native bone cannot be used for massive bone reconstruction, perfusion-decellularized massive bone should be compared with other surgical procedures (available for large defects), such as TCP scaffolds, to establish its place in the surgical algorithm.

## Supporting information

S1 File SD1: 3D-printed cutting guide used on the automatic precision cutting machine to obtain two parallel faces. On the left, the cutting guide with a silicon part in green to avoid damaging the specimen after fixing it with the two screws. On the right, the allograft fixed with two screws in the guide, which in turn is fixed to the cutting machine. **SD2-A. Irradiated specimens’ sizes. SD2-B. Non-Irradiated specimens sizes. SD2-C. Explanted specimens sizes.**
(DOCX)

S1 Checklist Plos One humane endpoints checklist. (DOCX)
