# Supplementary material for: Surgical mechanical properties of perfused decellularized massive bone allografts: An comparative in vivo test
Source: PLoS One. 2025 Jun 2;20(6):e0322901. doi: 10.1371/journal.pone.0322901 (PMC12129329; doi:10.1371/journal.pone.0322901)
Supplement: S1 File — On the left, the cutting guide with a silicon part in green to avoid damaging the specimen after fixing it with the two screws. On the right, the allograft fixed with two screws in the guide, which in turn is fixed to the cutting machine. SD2-A. Irradiated specimens’ sizes. SD2-B. Non-Irradiated specimens sizes. SD2-C. Explanted specimens sizes. (DOCX) [file pone.0322901.s001.docx]

**SUPPLEMENTARY DATA**

**SD1-A. Irradiated specimens’ sizes**

| Sample | Compression | | 3-Point Bending | |
| --- | --- | --- | --- | --- |
|  | Native | Decell | Native | Decell |
| F1 | $l_{0}=15.9$  $w_{0}=16.2$  $t_{0}=3.2$ | $l_{0}=17.3$  $w_{0}=11$  $t_{0}=4.5$ | $L_{0}=31.3$  $W_{0}=16.6$  $T_{0}=6.7$ | $L_{0}=33.1$  $W_{0}=14.3$  $T_{0}=7.5$ |
| F2 | $l_{0}=13.6$  $w_{0}=18.3$  $t_{0}=7.4$ | $l_{0}=15.2$  $w_{0}=20$  $t_{0}=8.2$ | $L_{0}=32.9$  $W_{0}=11.1$  $T_{0}=5.8$ | $L_{0}=27.3$  $W_{0}=9.1$  $T_{0}=4.7$ |
|  |  |  |  |  |
| F3 | $l_{0}=16.8$  $w_{0}=21.5$  $t_{0}=7.9$ | $l_{0}=19.3$  $w_{0}=19.2$  $t_{0}=6.7$ | $L_{0}=22.1$  $W_{0}=12.3$  $T_{0}=6.1$ | $L_{0}=20.4$  $W_{0}=12.2$  $T_{0}=7.4$ |
|  |  |  |  |  |
| F4 | $l_{0}=12.0$  $w_{0}=16.1$  $t_{0}=6.5$ | $l_{0}=11.6$  $w_{0}=8.1$  $t_{0}=3.2$ | $L_{0}=25.9$  $W_{0}=10.2$  $T_{0}=4.0$ | $L_{0}=22.9$  $W_{0}=11.1$  $T_{0}=6.2$ |
|  |  |  |  |  |
| F5 | $l_{0}=19.9$  $w_{0}=20.2$  $t_{0}=10.8$ | $l_{0}=11.8$  $w_{0}=11.7$  $t_{0}=6.4$ | $L_{0}=31.9$  $W_{0}=11.2$  $T_{0}=5.6$ | $L_{0}=23.5$  $W_{0}=12.4$  $T_{0}=4.3$ |
|  |  |  |  |  |
| F6 | $l_{0}=16.1$  $w_{0}=7.5$  $t_{0}=3$ | $l_{0}=14.1$  $w_{0}=16.8$  $t_{0}=5.4$ | $L_{0}=27.5$  $W_{0}=12.6$  $T_{0}=5.8$ | $L_{0}=27.5$  $W_{0}=8.6$  $T_{0}=3$ |
|  |  |  |  |  |
| F7 | $l_{0}=15.3$  $w_{0}=16.2$  $t_{0}=5.1$ | $l_{0}=12.6$  $w_{0}=8.2$  $t_{0}=4.4$ | $L_{0}=25.2$  $W_{0}=12.5$  $T_{0}=4$ | $L_{0}=17.1$  $W_{0}=8.5$  $T_{0}=4.2$ |
|  |  |  |  |  |
| F8 | $l_{0}=14.3$  $w_{0}=16.3$  $t_{0}=9.3$ | $l_{0}=10.8$  $w_{0}=10.74$  $t_{0}=6.64$ | $L_{0}=31.3$  $W_{0}=9.3$  $T_{0}=6.3$ | $L_{0}=24.8$  $W_{0}=10.0$  $T_{0}=5.2$ |

Lengths ($l_{0},L_{0}$), widths ($w_{0},W_{0}$) and thicknesses ($t_{0},T_{0}$) are given in mm. The dark gray specimens were not used as they were destroyed during preparation.

**SD1-B. Non-Irradiated specimens sizes**

| Sample | Compression | | Flexion 3 points | |
| --- | --- | --- | --- | --- |
|  | Native | Decell | Native | Decell |
| F1 | $l_{0}=15.8$  $w_{0}=16.9$  $t_{0}=7.2$ | $l_{0}=17$  $w_{0}=15.7$  $t_{0}=6.3$ | $L_{0}=31.0$  $W_{0}=15.0$  $T_{0}=7.2$ | $L_{0}=34.0$  $W_{0}=12.0$  $T_{0}=7.7$ |
| F2 | $l_{0}=19.4$  $w_{0}=17.4$  $t_{0}=7.2$ | $l_{0}=14.0$  $w_{0}=17.9$  $t_{0}=6.3$ | $L_{0}=34.9$  $W_{0}=13.6$  $T_{0}=5.9$ | $L_{0}=30.1$  $W_{0}=10.3$  $T_{0}=3.0$ |
| F3 | $l_{0}=12.3$  $w_{0}=18$  $t_{0}=7.1$ | $l_{0}=14.1$  $w_{0}=27.4$  $t_{0}=6.5$ | $L_{0}=24.6$  $W_{0}=12.3$  $T\_0=5.0$ | $L_{0}=22.8$  $W_{0}=10.6$  $T_{0}=5.0$ |
| F4 | $l_{0}=13.0$  $w_{0}=16.3$  $t_{0}=8.2$ | $l_{0}=10.8$  $w_{0}=16.1$  $t_{0}=6.1$ | $L_{0}=24.4$  $W_{0}=10.5$  $T_{0}=4.0$ | $L_{0}=17.7$  $W_{0}=9.2$  $T_{0}=4.3$ |
| F5 | $l_{0}=15.7$  $w_{0}=17.4$  $t_{0}=7.5$ | $l_{0}=11.7$  $w_{0}=11.2$  $t_{0}=5.5$ | $L_{0}=33.2$  $W_{0}=12.5$  $T_{0}=6.5$ | $L_{0}=22.2$  $W_{0}=11.2$  $T_{0}=7.2$ |
| F6 | $l_{0}=12.1$  $w_{0}=16.9$  $t_{0}=4.8$ | $l_{0}=12.3$  $w_{0}=18.6$  $t_{0}=7.6$ | $L_{0}=28.7$  $W_{0}=10.1$  $T_{0}=7.1$ | $L_{0}=26.3$  $W_{0}=11.5$  $T_{0}=3.8$ |
| F7 | $l_{0}=12.9$  $w_{0}=18.4$  $t_{0}=6.9$ | $l_{0}=11.1$  $w_{0}=10.0$  $t_{0}=4.8$ | $L_{0}=27.9$  $W_{0}=8.2$  $T_{0}=3.5$ | $L_{0}=23.1$  $W_{0}=10.9$  $T_{0}=5.6$ |
| F8 | $l_{0}=12.7$  $w_{0}=15.6$  $t_{0}=7.6$ | $l_{0}=13.0$  $w_{0}=16.1$  $t_{0}=9.7$ | $L_{0}=23.1$  $W_{0}=11.6$  $T_{0}=4.4$ | $L_{0}=23.5$  $W_{0}=9.8$  $T_{0}=4.5$ |

Lengths ($l_{0},L_{0}$), widths ($w_{0},W_{0}$) and thicknesses ($t_{0},T_{0}$) are given in mm.

**SD1-C. Explanted specimens sizes**

| Sample | Compression | | Flexion 3 points | |
| --- | --- | --- | --- | --- |
|  | native | Decell | Native | Decell |
| 331 | $l_{0}=15.4$  $w_{0}=28.2$  $t_{0}=12.8$ | $l_{0}=15.3$  $w_{0}=25.6$  $t_{0}=15.1$ | $L_{0}=23.6$  $W_{0}=10.5$  $T_{0}=8.1$ | $L_{0}=17.3$  $W_{0}=16.1$  $T_{0}=11.0$ |
|  |  |  |  |  |
| 336 | $l_{0}=11.6$  $w_{0}=16.9$  $t_{0}=10.4$ | $l_{0}=15.8$  $w_{0}=18.7$  $t_{0}=9.5$ | $L_{0}=14.9$  $W_{0}=12.0$  $T_{0}=8.0$ | $L_{0}=26.1$  $W_{0}=12.1$  $T_{0}=7.6$ |
|  |  |  |  |  |
| 523 | $l_{0}=16.4$  $w_{0}=17.6$  $t_{0}=11.0$ | $l_{0}=12.8$  $w_{0}=17.0$  $t_{0}=7.07$ | $L_{0}=28.6$  $W_{0}=8.3$  $T_{0}=3.9$ | $L_{0}=19.2$  $W_{0}=9.5$  $T_{0}=5.9$ |

Lengths ($l_{0},L_{0}$), widths ($w_{0},W_{0}$) and thicknesses ($t_{0},T_{0}$) are given in mm.

**SD2: 3D-printed cutting guide used on the automatic precision cutting machine to obtain two parallel faces**. On the left, the cutting guide with a silicon part in green to avoid damaging the specimen after fixing it with the two screws. On the right, the allograft fixed with two screws in the guide, which in turn is fixed to the cutting machine.
